# A survey of the beliefs regarding international emergency medicine among fourth-year medical students planning on matching in emergency medicine

**DOI:** 10.1186/1865-1380-6-18

**Published:** 2013-06-20

**Authors:** Elissa M Schechter-Perkins, Nicolas P Forget, William K Mallon

**Affiliations:** 1 Boston University, Boston, MA, USA; 2 Vanderbilt University, Nashville, TN, USA; 3 University of Southern California, Keck School of Medicine, Los Angeles, CA, USA

**Keywords:** International emergency medicine, Medical students, Residency, Emergency medicine education, Cross-sectional survey

## Abstract

**Background:**

With the recent growth of fellowships in international emergency medicine, the authors sought to evaluate medical students’ attitudes toward international emergency medicine and to determine the effects these attitudes have on their residency selection.

**Methods:**

Study design: Cross-sectional survey. Data collection: An anonymous, eight-question online survey was distributed to all members of the American Academy of Emergency Medicine Resident and Student section. This survey was also distributed to fourth-year medical students rotating through the Emergency Department at Los Angeles County and the University of Southern California.

**Results:**

Ninety-eight surveys were collected, 61 from rotating students and 37 from the AAEM mailing. There were no statistically significant differences in responses between the two groups. Of the respondents, 49.4% of have been exposed to IEM, and 46.9% have participated in international health projects. Ninety-four percent agree that IEM is an exciting career option. Seventy-nine percent said programs with IEM opportunities are more appealing than those without, and 45% said the presence of IEM opportunities would be an important factor in rank list; 53% believe that IEM requires formal public health training, and 63% believe it requires tropical medicine training; 68.3%of respondents speak a language in addition to English. This subset was more likely to have participated in IEM projects previously (*p* = 0.026) but not more likely to make match choices based on IEM.

**Conclusions:**

Half of medical students surveyed had prior experience in international health, and most agree that international emergency medicine is an exciting career option. Over two thirds believe that the presence of IEM opportunities will be a factor in their match list decision.

## Background

International emergency medicine (IEM) has experienced a growth in recognition in the US over the past 10 years. This is exemplified by the formation of interest groups among major emergency medicine (EM) bodies and the creation of new fellowships for physicians interested in specialized post-graduate training [1]. This growth may be attributed in part to the success of Emergency Medicine in the US (US) and around the developed world. As low- and middle-income countries (LMIC) strive to modernize their health-care systems, they turn to trained emergency experts for assistance. In addition, international humanitarian organizations recognize the value of Emergency Physicians in the field as physicians with a broad knowledge base who are adaptable and able to work in stressful situations with limited resources [2]. The number of countries hosting formal EM residency training continues to expand rapidly.

There are now 42 international emergency medicine fellowships across the US [3], a tremendous growth from 2004 when only 8 such programs were listed [4]. Clearly, the efforts of the leaders who initiated and grew the fellowships have paid dividends [5]. In fact, interest in IEM is growing among other countries with mature EM systems, and fellowships are likely to start there too [6]. Other evidence of the growth of IEM includes the recent creation of the Global Emergency Medicine Academy under the auspices of the Society for Academic Medicine (SAEM). At the Academy of Emergency Physicians (ACEP), the IEM interest section is one of the largest with over 1,000 members [7].

As international emergency medicine grows as a specialty, opportunities for residents to acquire IEM skills during their EM training grows as well. Residents may have the opportunity to take part in rotations abroad, interact with international colleagues studying in US EDs and even take part in didactic programs focusing on IEM methods.

The role of IEM opportunities in the selection of an emergency medicine residency program for fourth-year medical students was evaluated in 2002 [8]. Given the explosion of opportunities during residency as well as fellowships to promote leadership in the field, a re- evaluation of medical student’s interest in the field was needed. Specifically, we aimed to expand some of the questions of Dr. Dey’s group to assess whether medical students’ past experiences in international health, their language skills, their views of and attitudes toward international emergency medicine, as well as their interest in a potential career in international emergency medicine and whether these had an effect on their residency selection.

## Methods

Study Design: This was a cross-sectional survey to evaluate the influence of international EM opportunities on fourth-year medical students planning on applying to EM residency programs. It was reviewed by the Internal Review Board (IRB) at the University of Southern California and given exempt status, and all research activities were in compliance with the Helsinki Declaration. Data collection: In June 2007, an anonymous, eight-question online survey was developed by the authors and was distributed via an email link (surveymonkey.com) to all members of the American Academy of Emergency Medicine Resident and Student section. This survey was also distributed to fourth-year medical students rotating through the Department of Emergency Medicine at Los Angeles County and the University of Southern California, who indicated they were planning on matching in Emergency Medicine, during the 2007–2008 academic year.

Data analysis: Data was analyzed with SPSS 15.0. Likert-type scale scores were treated as interval data and analyzed with descriptive statistics and one-sample Student’s *t* tests, then converted to binary scores for comparison with *χ*2 tests for homogeneity. Surveys with incomplete responses were included in the analysis.

## Results

Ninety-eight surveys were collected, 61 from rotating students and 37 from the AAEM email solicitation. There were no statistically significant differences in responses to any question between the two groups. Demographics of respondents are described in Table 1.

**Table 1:** Survey results

**Age**	
Under 26 years	7%
26–30 years	46%
3135 –years	7%
3640 –years	2%
41 years and older	1%
Gender	
Female	43%
I have been exposed to IEM	49.4%
I have participated in international health projects in the past	46.9%
I have lived in a foreign country in the past	34.7%
I possess sufficient language capabilities to practice EM in foreign countries where English is not spoken	68.3%
IEM is an exciting career option for EPs	
Strongly agree or somewhat agree	84 (94%)
Neutral/somewhat disagree or strongly disagree	5 (6%)
Programs that provide international opportunities (including	
elective rotations abroad) to residents are more appealing to me than those that do not	
Strongly agree or somewhat agree	74 (79%)
Neutral/somewhat disagree or strongly disagree	20 (21%)
The presence or absence of international opportunities will be an important factor in my ranking of EM programs for the match	
Strongly agree or somewhat agree	42 (45%)
Neutral/somewhat disagree or strongly disagree	51 (55%)
International medicine and public health should be the domain of government agencies and NGOs rather than emergency physicians	
Strongly agree or somewhat agree	13 (14%)
Neutral/somewhat disagree or strongly disagree	81 (86%)
International emergency medicine requires formal tropical medicine training	
Strongly agree or somewhat agree	59 (63%)
Neutral/somewhat disagree or strongly disagree	35 (37%)
International emergency medicine requires formal public health training	
Strongly agree or somewhat agree	49 (53%)
Neutral/somewhat disagree or strongly disagree	43 (47%)

Nearly half (43/87, 49.4%) of respondents have been exposed to IEM, and 46/88 (52.3%) have participated in international health projects. An overwhelming majority (83/85, 97.6%) agree IEM is an exciting career option. Many (74/79, 93.7%) said residency programs with IEM opportunities are more appealing than those without, and 42/61 (68.9%) said the presence of IEM opportunities would be a factor in their rank list.

A majority (49/73, 67.1%) feel IEM requires formal public health education, and 59/80 (73.8%) feel it requires tropical medicine training.

Of the respondents, 56/82 (68.3%) speak at least one language in addition to English, and for the majority [48/56 (85.7%)] one of these languages is Spanish. The subset of respondents who speak additional languages was more likely to have participated in IEM projects previously (OR 3.17, 95% CI 1.13-8.91) but not more likely to make match choices based on IEM (OR 2.43, 95% CI 0.76-7.7). The subset of students who have previously participated in international health projects did not indicate they would find programs with IEM opportunities more appealing (OR 0.9, 95% CI 0.80-1.01), but were more likely consider these opportunities as a factor in their match list (OR 4.06, 95% CI 1.11-14.80).

## Discussion

The results of this survey are very encouraging for those involved in international emergency medicine. The increase in the number of fellowships indicates that many of these medical students are pursuing advanced international work beyond what may be available during residency training.

When compared to prior research [8], our data are generally consistent with a strong measure of enthusiasm for IEM. Our respondents feel strongly that additional training is required to perform IEM (such as advanced training in either public health or tropical medicine), perhaps mirroring the trend in increased fellowships. This suggests that medical students understand the professionalization of IEM and the value of academic degrees in order to carry out a project effectively. We found approximately half of the respondents have had some international medicine exposure in the past, and it is notable that these particular respondents would consider future opportunities important in their matching. Medical student’s language skills predicted prior work in the field, but did not predict he or she would rank a residency program with IEM opportunities more favorably. This lack of association may be due to the relatively small sample size.

Compared to prior research, this survey indicates medical students still find IEM opportunities to be an important factor in the ranking of residency programs [8].

Two recent studies looked at factors affecting the rank list for applicants to emergency medicine. One found a balance of geographic location and program characteristics factors into the residency decision. [9]. The second found the most influential variables in residency choice are the institutional and residency program director’s reputation and hospital facilities [10]. However, IEM was not specifically asked about in either study, and as a result interest in the field was not considered. This was cited as a weakness of the latter study.

Recent data suggest medical students generally have a strong interest in global health. Our research indicates emergency medicine generates a very strong interest and is likely both a driving factor and result of the development of IEM as a subspecialty of EM.

### Limitations

One limitation of this study, as with all surveys, was the self-reporting nature of the data. In addition, our study was performed prior to the respondents’ rank list such that their preferences given may not be reflective of the final decisions made on their actual rank list. Furthermore, we have a small sample size. Another limitation of this study is related to potential selection bias introduced via the sampling methods used. Most survey recipients were captured during a rotation at a program with an active IEM department including a fellowship and a long history in the field, so the decision to rotate in this setting may reflect interest in IEM not found in the overall EM applicant pool. We supplemented our research by querying fourth-year medical students who were members of the American Academy of Emergency Medicine Residents and Students section, but did not obtain as many responses from this recruitment method. Additionally, student members of this group may be more proactive about their involvement in EM in general than non- members. Finally, a limitation is the data are 6 years old, and thoughts and perspectives may have changed since this survey was administered.

## Conclusion

International emergency medicine appears to be an important factor in medical students’ ranking of emergency medicine residency programs. No factor has emerged as a reliable predictor of this interest except for a basic interest and enthusiasm for this growing subspecialty.

## Competing interests

The authors declare that they have no competing interests.

## Authors’ contribution

ESP, NF, and WM conceived the study and designed the trial. ESP and NF undertook recruitment, supervised data collection, managed data, and performed data analysis. ESP, NF, and WM interpreted the data and contributed to the manuscript. All authors read and approved the final manuscript.
